# The Impacts of Female Access during Rearing on the Reproductive Behavior and Physiology of Pekin Drakes, and Flock Fertility

**DOI:** 10.3390/ani12212979

**Published:** 2022-10-29

**Authors:** Lindsey J. Broadus, Brian Lee, Maja M. Makagon

**Affiliations:** 1 Animal Behavior Graduate Group, College of Biological Sciences, University of California, Davis, CA 95616, USA; 2 Center for Animal Welfare, Department of Animal Science, College of Agricultural and Environmental Sciences, University of California, Davis, CA 95616, USA; 3 Maple Leaf Farms, Inc., Leesburg, IN 46538, USA

**Keywords:** behavior, testosterone, fertility, Pekin duck, physiology, reproduction

## Abstract

**Simple Summary:**

Male and female ducklings are typically reared in same-sex groups. With the goal of improving the males’ reproductive performance, and overall flock fertility, some flock owners place several female ducklings into the otherwise all-male pens during rearing. However, the relationships between rearing, drake reproductive success, and flock fertility have not been confirmed. To fill these knowledge gaps, we compared the frequencies of correctly oriented mounts and circulating testosterone levels of drakes reared with and without physical access to females, and the impacts of these rearing treatments on flock fertility. Rearing treatment did not impact any of the measured variables; however, all were affected by age. Individual variation in behavior and testosterone measures were noted in both treatment groups. We conclude that rearing male ducklings with auditory and visual, but without physical access to female ducklings is sufficient for promoting reproductive behavior and physiology, and securing high fertility within this Pekin duck breed.

**Abstract:**

Commercially housed Pekin ducks (*Anas platyrhynchos*) are typically reared in same sex groups to facilitate separate diet provisioning. Several female ducklings are sometimes mixed into the otherwise all-male pens. This practice is thought to increase flock reproductive success. To evaluate this hypothesis, we reared ducklings in alternating same-sex groups (150 hens or 30 drakes/pen; 8 groups/sex) and evaluated the impacts of rearing on drake mounting behavior, testosterone levels, and flock fertility. At 12 days, three females were placed into four of the male duckling pens. At 20–22 weeks of age, adjacent male and female pens were moved into pens within a breeder barn, and combined to form mixed-sex pens. The number of correctly aligned mounts performed by 10 focal drakes per pen was evaluated over 3 days (12 h/day) at 26, 32, and 45 weeks of age. Circulating testosterone concentrations were analyzed from blood plasma samples collected from the focal drakes at 15 (baseline), 22, 28, 34 and 45 weeks of age. Pen-level fertility was determined at 33–34 and 45–46 weeks of age. Mount and testosterone data were analyzed using a Generalized Linear Mixed Model and a Linear Mixed Model in R 4.0.5, with duck in pen as a random effect. A Linear Mixed Model was used to analyze fertility data, with pen as a random effect. None of the measured variables were impacted by rearing treatment, but all varied with flock age. Physical access to female ducklings during rearing did not enhance flock reproductive success.

## 1. Introduction

In commercial settings for breeder Pekin ducks, females and males are often reared separately to account for differences in early diet provisioning. Some farms rear male ducklings with physical access to female ducklings, also referred to as ‘imprinting’ hens (often 7–10% females in the group) [1]. This management strategy is maintained to encourage duck reproductive success in adulthood. However, the relationship between rearing and fertility has not been confirmed, and the proximate mechanisms that might link the early rearing environment with adult duck reproduction are not well understood. We aimed to fill these knowledge gaps.

Potential differences in fertility could be due to differences in sexual behaviors developed by drakes that had physical access to females as ducklings and those that did not [1]. Influences from physical exposure to the opposite sex, particularly in the early social environment, can have lasting effects on the expression of social behaviors [2]. Among domesticated species, White Leghorn roosters that had been reared with physical access to female chicks showed increased duration and frequency of mounting and mating behaviors as compared to males reared without such physical access. These differences subsided as the birds aged. The number of mating attempts and successful copulations declined over time for both groups [3]. It is possible that rearing male ducklings with physical access to female ducklings may similarly result in increased mounting behavior, particularly after the female and male groups are initially mixed.

Variation in fertility and reproductive success in ducks could also be attributed to differences in male testosterone levels [4,5,6,7]. A relationship between testosterone levels and the performance of drake sexual behaviors has been well documented in ducks and other seasonally breeding birds [8,9,10,11,12,13,14]. As described by the Challenge Hypothesis [15,16] testosterone may have continuous activational effects in sexually mature drakes, but is likely to act organizationally in the early environment. In turn, changes in interactions with the opposite sex often provide feedback on the endocrine system which affects circulating testosterone levels [17]. Higher testosterone levels are suggested to be closely related to increased fertility and reproductive success in ducks [4,5,6,7].

We aimed to evaluate the assumed relationship between the practice of raising male ducklings with ‘imprinting’ female ducklings and flock fertility and possible underlying proximate mechanisms. We predicted that drakes that had been reared with female ducklings would display higher frequencies of mounting behavior and have higher circulating testosterone levels as compared to males reared in all-male groups, and that flock fertility would be higher among flocks that utilized ‘imprinting’ females during rearing.

## 2. Materials and Methods

### 2.1. Animals and Housing

Day-old Pekin ducklings (N = 1440) were distributed across eight two-pen units within a single developer barn located on a breeder duck farm. The units were separated from one another by an empty pen, and served as treatment replicates. Within each unit, 150 female ducklings were placed into the larger pen (15.24 m × 6.10 m), and 30 male ducklings were placed into the smaller pen (15.24 m × 3.05 m). All birds had visual access to ducklings of the opposite sex through the mesh fence that separated adjacent pens. At 12 days of age, three female ducklings were moved into every other all-male pen from the adjacent all-female pen. Thus, male ducklings in four pens were raised with direct physical contact with female ducklings, and male ducklings in the remaining four pens had only visual, auditory, and olfactory exposure to female ducklings through the mesh fence. Six sets of adjacent female and male pens were combined and transferred into the breeder barn at 20 weeks of age. The female and male groups from the remaining two sets of pens (one per treatment) were combined and moved to the breeder barn at 22 weeks of age due to farm staffing constraints. Ten randomly selected focal drakes per pen were individually color marked across their back at the time of transfer. In total, the breeder house contained four pens of mixed sex ducks (180/pen) from each of the two rearing treatments. To account for effects of location, pens containing each treatment were alternated within the breeder barn, as was the case in the developer barn.

### 2.2. Behavioral Analysis

**Figure 1:**
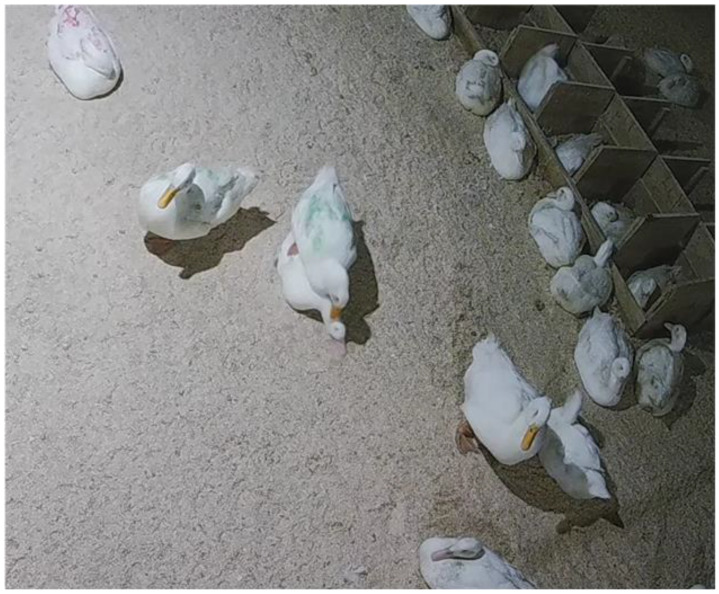
A marked focal Pekin drake performs a correctly oriented mount on top of a hen. The orientation is head-to-head and tail-to-tail.

Focal drake behavior was transcribed from video recorded over three consecutive days when the ducks were 26, 32, and 45 weeks old. We selected these time points based on their relevance to duck egg production: Eggs were first collected to be set at 26 weeks of age, 32 weeks of age corresponded to peak lay, and 45 weeks of age was when egg production begins to plateau. As part of standard management procedures, some ducks were removed from the flock due to injury or mortality, which impacted the number of focal birds available. In total, 39 ducks per treatment were observed at 26 weeks of age, 38 ducks from the same sex treatment and 37 ducks for the mixed sex treatment were observed at 32 weeks of age, and 36 ducks from the same sex treatment and 35 ducks for the mixed sex treatment were observed at 45 weeks of age. We used continuous sampling strategy to tabulate all instances of female-directed correctly oriented mounts performed by each focal drake from 06:00–18:00 h on each of the three consecutive days recorded at each time point (36 h video/time point). Correctly oriented mounts were defined as mounts with parallel head-to-head and tail-to-tail orientation that lasted for at least one second (Figure 1). This mounting orientation opens the potential for copulation to occur due to the ballistic nature of the drake’s penis. Multiple observers collected behavioral data. Inter-rater reliability was confirmed based on two hours of footage per age, with 90% accuracy compared to the most experienced observer (L. Broadus).

### 2.3. Blood Sampling and Hormone Analysis

Blood samples were collected from the same individually marked drakes between 09:00 and 11:00 h at each of the five time points. A baseline sample was collected from 10 drakes per pen (N = 80 drakes total) at 15 weeks of age, before reproductive maturity. Additional samples were taken shortly after reproductive maturity was reached (22 weeks of age), when collection of fertile eggs for incubation and hatching commenced at the farm (28 weeks of age), near peak production (34 weeks of age), and after egg production had plateaued (45 weeks of age). As was the case for behavioral observations, 39 ducks per treatment were sampled at 22 weeks of age. The same ducks were also sampled at 28 weeks of age. At 34 weeks of age, 38 and 37 ducks were sampled from the same sex and mixed sex rearing treatment groups, respectively. The final blood collection included 71 ducks after an additional two focal ducks were removed from each treatment group.

At each time point, we drew 2 mL blood from the metatarsal vein using 21-G needles and 3cc syringes. Blood was immediately stored in vacutainers on ice in a cooler for transportation to University of California, Davis, where the samples were spun for 15 min at 1500 RPM. Plasma was siphoned off into duplicate cryovials and stored at −20 °C until analyzed by competitive ELISA (Cayman Chemical, Ann Arbor, MI, USA). The ELISA kit had been previously used for serum testosterone levels in the same genetic strain of Pekin duck [18]. Following protocols outlined by Love and Williams [19] and Steenweg [20], a modified protocol was utilized for validation. The protocol from the kit was followed, except plates were incubated at 26 °C while shaking at 500 rpm. Samples were run in duplicate on a 96-well plate following a serial dilution protocol that replicated the standard curve dilutions of the buffer stock solution (1:8 for plasma samples taken before reproductive maturity and 1:64 for breeding age plasma samples). Each plate included a standard curve and duplicates of a control pool sample of Pekin drake plasma. Absorbance was read at 405 nm, and the readings were analyzed using a Cayman-provided excel worksheet. The inter-assay coefficient of variation across all plates calculated based on pooled plasma was 3.36%. The intra-assay coefficient was 8.18%.

### 2.4. Egg Production Data 

Egg fertility data obtained from flock records provided by Maple Leaf Farms, Inc. Hatchery (San Joaquin Valley, CA, USA). Eggs were considered fertile when containing an embryo as determined by candling. To determine impacts of age on fertility and facilitate comparisons with plasma testosterone levels, fertility data were averaged by pen from the weeks of 33–34 weeks of age (January 2019) and 45–46 weeks of age (April 2019).

### 2.5. Statistical Analysis

At the individual level, a Generalized Linear Mixed Model (GLMM) was used to assess the effect of the rearing treatment (mixed or same sex) on correctly oriented mounts (response variable) in R 4.0.5 (glmmTMB package) [21,22]. The response variable was compared to the same sex and mixed rearing treatments (explanatory variables-fixed effects). In addition to treatment, flock age, and interactions between treatment and age were considered fixed effects. Pen was accounted for as a random effect, with individual nested in pen. Backwards selection was used on the model to determine best fit, and the most parsimonious model was selected. The count data fit a zero inflated negative binomial model. The criteria for normality of residuals was met based on a Quantile-Quantile plot that included a Kolmogorov–Smirnov Test and dispersion test, along with a within group deviation from uniformity and a Levene Test for homogeneity of variance using the DHARMa package [23] in R 4.0.5. An analysis of variance (ANOVA) table was created to attain an analysis of deviance. To understand the output of a model with a negative binomial regression, type II Wald chi-square tests were performed.

To assess the effect of the treatment (same or mixed sex rearing) on drake testosterone levels and pen-level fertility, Linear Mixed Models (LMM) were used (LMER in lme4 package) [24] in R 4.0.5 [22]. Testosterone levels and fertility percentages were log-transformed for normality, which was assessed based on evaluation Q-Q and residual vs. fitted values plots and homoscedasticity of the independent variables. Thus, for the first model, log-transformed testosterone levels were the response variable. For the second model, log-transformed averaged fertility percentages at 33–34 weeks of age and 45–46 weeks of age were the response variable. For both models, treatment and age were both considered fixed effects (explanatory variables). For the model analyzing testosterone levels, an interaction between treatment and age was also included as a fixed effect. Individual focal drake nested in pen (model 1) or pen (model 2) were considered as random effects. Backward stepwise selection using ANOVA allowed for model comparison. A *p*-value of <0.05 was considered statistically significant. Testosterone levels and fertility percentages were back-transformed for reporting means and standard errors and for use in figures. We relied on nonparametric statistics by utilizing Spearman’s rank correlation coefficient (cor.test function in R) to determine the relationship between testosterone levels and fertility, averaged at the pen level. All plots were created using the ggplot2 package [25].

## 3. Results

### 3.1. Behavioral Data

**Figure 2:**
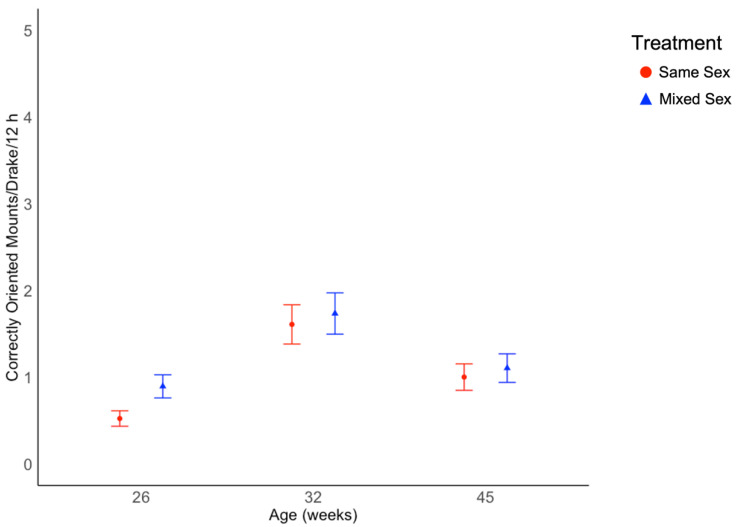
Average number of correctly oriented mounts for Pekin drakes over three 12 h observation periods at 26, 32, and 45 weeks of age for same sex rearing groups and mixed sex rearing groups. Mount rate values differed by age (*p* < 0.001) but not treatment. Mount rate values are presented as estimated marginal means and standard errors.

**Figure 3:**
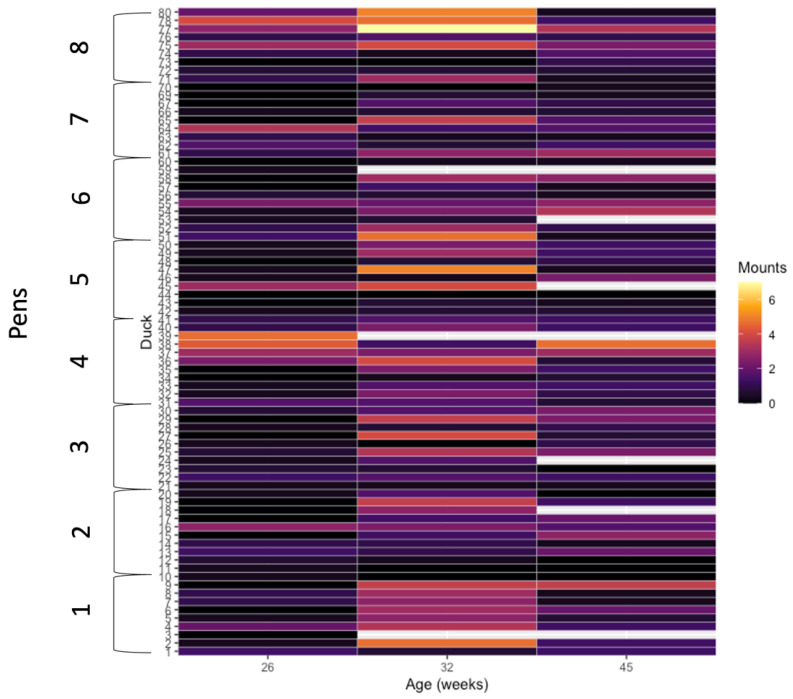
Number of correctly oriented mounts for individual Pekin drakes over three 12 h observation periods at 16, 32, and 45 weeks of age.

Frequencies of correctly oriented mounting events performed by focal drakes were not affected by rearing treatment (χ^2^ = 1.26, df = 1, *p* = 0.261; Figure 2), but were affected by age (χ^2^ = 76.1, df = 2, *p* < 0.001; Figure 2; Supplementary Table S1). The interaction of treatment and age was not significant (χ^2^ = 5.77, df = 2, *p* = 0.056). Mounting events increased from 26 to 32 weeks of age, then decreased to 45 weeks of age in both rearing treatment groups. Individual focal drakes varied in their frequency of mounting behavior. The widest range in variation of individual mounting rates was observed at 32 weeks of age, with 0–10 events for individual drakes with the lowest and highest rates, respectively, within a 12 h period (Figure 3).

### 3.2. Hormone Data

**Figure 4:**
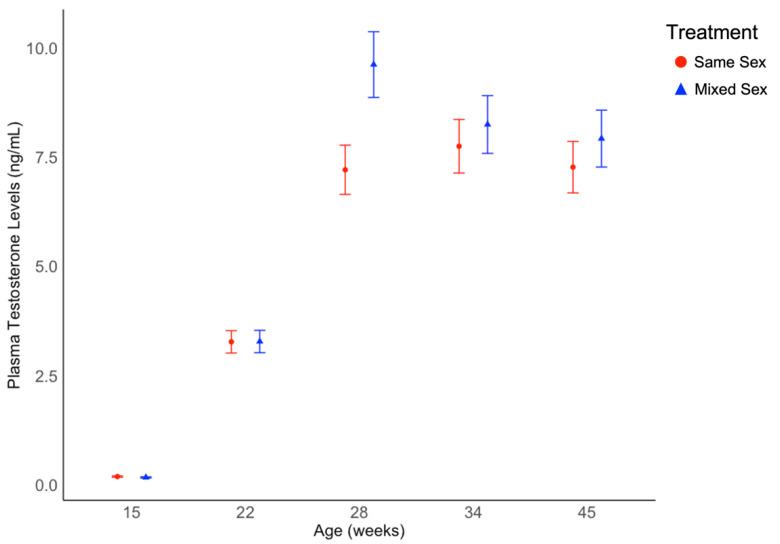
Circulating average testosterone concentrations in drake Pekin ducks from same sex rearing groups and mixed sex rearing groups from 15 to 45 weeks of age. Testosterone levels differed by age (*p* < 0.001) but not treatment. Testosterone concentration values are back-transformed from a logarithmic transformation and presented as estimated marginal means and standard errors.

As shown in Figure 4 testosterone levels were affected by age (χ^2^ = 4179, df = 4, *p* < 0.001), but not treatment (χ^2^ = 1.04, df = 1, *p* = 0.307, data shown in Supplementary Table S2), or the interaction between treatment and age (χ^2^ = 8.25, df = 4, *p* = 0.083). A distinct increase was observed between model-estimated marginal mean concentrations at 15 weeks of age and 22 weeks of age. A numerical increase in mean testosterone concentrations was also observed between 22 and 28 weeks of age for both treatment groups. Between 28, 34, and 45 weeks of age, levels slightly increased then decreased for the same sex treatment and slightly decreased for the mixed sex treatment. Pronounced individual variation was observed in circulating testosterone levels (ex. 1.31 ng/mL–16.83 ng/mL at 34 weeks of age; Figure 4), except at the baseline time point (15 weeks of age).

### 3.3. Fertility Data

**Figure 5:**
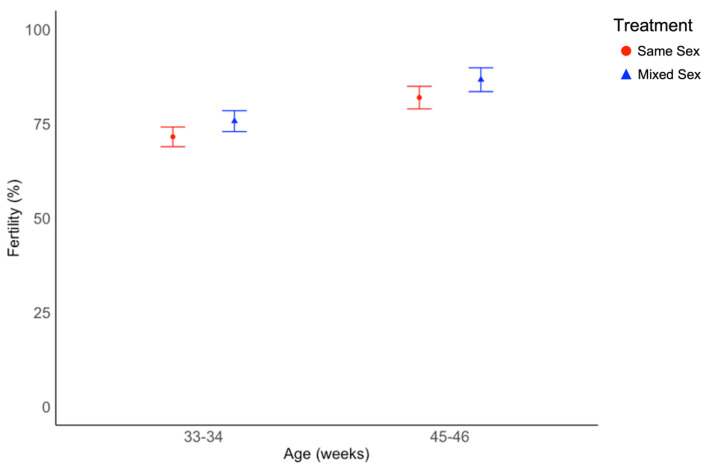
Average fertility percentages calculated based on pen-level data collected when ducks were 33 and 34, and 45 and 46 week of age. Average flock fertility differed by age (*p* < 0.001), but not treatment. Fertility values are back-transformed from a logarithmic transformation and presented as estimated marginal means and standard errors. Fertility was determined based on candling of eggs.

The percentage of fertile eggs was similar in the two treatments (χ^2^ = 1.42, df = 1, *p* = 0.233, Figure 5) but varied with age (χ^2^ = 23.5, df = 1, *p* < 0.001, Figure 5, Supplementary Table S3). Spearman’s rank correlation test was used to evaluate the relationship between fertility data collected from pens and drake testosterone levels averaged by pen, and found no correlation between the two variables (*p* = 0.223).

## 4. Discussion

We aimed to determine whether direct physical access to female ducklings of the same age during rearing influenced Pekin drake mounting behavior, circulating testosterone levels and flock fertility in adulthood. All of the measured variables were affected by age, but not rearing treatment. Other sensory cues from female ducklings experienced in the early rearing environment may have been sufficient to promote the development of drake physiological responses to hens later in life. Birds are known to rely on visual and auditory cues during social interactions [8], and all ducks, regardless of treatment, could see, hear, and smell ducks in adjacent pens. Additionally, the stimulation drakes experienced after they were mixed with hens may have been sufficient to incite appropriate reproductive behavior and testosterone production. Hen social cues have been shown to influence endocrine physiology in Mallard drakes; testosterone levels are higher in drake groups that experience incitement displays by hens than groups that do not [26].

The practice of adding female ducklings into otherwise all-male duckling pens in commercial flocks is done with the goal of promoting sexual imprinting [1]. It is well documented that early exposure to social cues provided during a critical period of development can lead to imprinting [27,28,29]. Sexual imprinting, specifically, occurs when a bird learns about the social environment and connects the learned information to sexual behavior, which is then stabilized [30]. However, whether sexual imprinting happens in mixed-sex reared duckling flocks, which exact cues are crucial for sexual imprinting to occur, and the long-term effects of sexual imprinting in this system are unknown. Our findings suggest that if sexual imprinting affected the development of drake sexual behavior during rearing, it might have been facilitated through visual, auditory, and/or olfactory cues, either in conjunction with or instead of haptic. Visual and auditory cues are known to be important factors involved in behavioral development in Pekin ducks [31]. Characteristics of sexual behaviors may alternately, or additionally, have developed and refined continually into adulthood [32,33,34]. In the context of this study, drakes may have gained sufficient experience after being mixed with females in the breeder barn, as the heavily female-skewed sex ratios would have facilitated access to many females. On the other hand, if imprinting did not occur with the addition of female ducklings into all-male groups, this could explain the lack of differences in testosterone levels and fertility between treatments.

Age related differences in mounting behavior and drake testosterone levels followed biologically relevant patterns. Modern strains of Pekin ducks reach sexual maturity by 26 weeks of age, with peak lay reported by approximately 32 weeks of age [1]. Accordingly, testosterone levels were low before sexual maturity and increased through 28 weeks of age. Mounting behavior was highest around peak lay, following a pattern previously reported for White Leghorn chickens [3]. In addition to age effects, the time of year could have contributed to changes in mounting frequencies and testosterone concentrations observed over time. Light and changes in temperature are known to facilitate reproductive activity in Pekin ducks, which are domesticated from the seasonally breeding Mallard [1]. Although ducks received supplemental artificial lighting, as is standard practice on breeding duck farms [1,35], the curtain sided barn used in our study also provided natural light. When exposed to natural light conditions, Pekin ducks show cycles of reproductive activity similar to Mallards [36,37]. In this study, natural day length ranged from approximately 9 h 39 min for the shortest day length in December 2018, at 28 weeks of age, to 13 h 15 min for the longest day length in April 2019, at 45 weeks of age [38]. Testosterone levels in ducks have previously been associated with diel and annual patterns. An increase in day length stimulates activation of the hypothalamic-pituitary-gonad (HPG) axis in Mallards [1,39], and has been shown to impact circulating testosterone levels in commercial Pekin drakes [39]. We were not able to separate the effects of age, from social or environmental impacts, including seasonal changes, as this was outside the scope of our study. However, it is likely that all of these factors contributed to observed changes in behavior and testosterone levels. When domestic Rouen ducks were placed under entirely natural conditions, drake testosterone levels fluctuated with seasonal variations influenced by both environmental and social factors [12], as predicted by the Challenge Hypothesis [15,16]. The Rouen ducks exhibited peak testosterone levels in October, after the refractory period, in December, when drakes increase social displays, and in March–April, when the breeding season began [12]. An earlier laboratory study [40] revealed instances where Pekin drakes displayed slight peaks in testosterone levels in September–October and December–January, with the most pronounced elevation in March and April [11]. Similar findings were reported by Assenmacher et al. [36], who also noted a pronounced peak in plasma testosterone levels starting in March in Pekin drakes housed under natural lighting conditions outdoors. Thus, entrained endogenous rhythms and natural environmental cues likely factor into Pekin duck breeding activity, in addition to the supplemental cues provided in the commercial setting. Investigating breeder housing light supplementation across all seasons in different commercial locations will reveal important information on how testosterone levels change in relation to multiple environmental and endogenous factors.

We observed individual variation in the frequency of correctly oriented mounting behavior and circulating testosterone levels across multiple ages. While one drake properly mounted hens ten times in a single day, others did not mount any females in the same span of time. Informally, we noted that some drakes did not attempt to mount females at all, while others were prevented from mounting by flockmates. Recent work by Ouyang et al. [41] revealed that metabolic differences might affect gene expression and signaling pathways important for sexual behaviors and other reproductive traits, thereby contributing to differences in Pekin drake sexual activity. Additionally, variation in mounts could also be attributed to hen receptivity. In Pekin ducks, mounting and fertilization may be prohibited when the female is not receptive. Female genital morphology is thought to reduce forced copulation in Muscovy ducks [42]. Though we could not observe copulation directly, we did informally annotate instances where female ducks did not assume the receptive posture [43], hindering mounting attempts by focal drakes, as well as instances of flockmates prohibiting drakes from mounting. Research on wild mallards found that mates will often prevent forced copulation from other drakes [44]. Pekin ducks do form pairs under natural conditions [45], however, it is not clear whether they do so in heavily female-skewed commercial settings. Thus, social dynamics and potential partner preferences could have affected observed frequencies of correctly oriented mounts by individual drakes.

Across free-living bird species, individual variation in circulating testosterone is affected by differences in both individual quality and environment [46]. In Mallards, drake testosterone levels under conditions with equal sex ratios did not differ between paired and unpaired drakes in the spring (during breeding) and fall, but paired drakes experienced a temporary decrease in testosterone levels after initial pair formation in the winter. When populations were male-skewed, paired drakes had higher testosterone levels than unpaired drakes during the breeding season [44]. As previously noted, it is unknown whether the ducks in this study paired up, making it difficult to evaluate whether pair formation could have contributed to the pronounced individual variation in testosterone levels. Social experiences prior to sampling may also have influenced testosterone levels. In sheathbills, testosterone levels were found higher among males that had just performed mutual pair displays than other males, and testosterone was highest in the male that had been observed copulating within 10 min of sampling [47]. Finally, although blood was quickly collected from focal individuals in each pen during our study, ducks in other pens may have been disturbed, which may have impacted their circulating testosterone levels. However, this was not likely to have a major influence, as the first pen sampled did not have ducks with the lowest average testosterone levels (data not reported). Thus, individual variation in Pekin drake testosterone levels observed in this study may be explained by a myriad of factors. Other studies have also noted individual variation in testosterone levels of sexually mature Pekin drakes. While direct comparison of data is not possible due to differences in study design and data collection protocols, reported average testosterone levels seem to be within a similar range to those reported in our study where ducks always had supplemental lighting (e.g., approximately 9 ng/mL in the winter with augmented light and approximately 18 ng/mL for ducks in the summer; Figure 4B in [39]).

Early access to physical contact with female ducklings did not impact flock fertility. Furthermore, there was no correlation between flock level fertility percentages and drake testosterone levels. Although drake testosterone levels can influence drake reproductive success, post-copulatory female choice in Pekin hens may greatly dictate which drakes fertilize eggs and the overall fertility levels [48]. In captive Mallards, offspring were sired less often when forced copulations with drakes that were not paired occurred [5,49]. To our knowledge, the impacts of hen choice on flock fertility have not been examined in commercial settings. Such studies should be explored, as the female-skewed sex ratios in this setting are particularly unique, and findings may yield novel basic and applied information related to flock reproductive success.

Flock level fertility percentages varied between the two biologically relevant ages studied. As previously discussed, both age and seasonality are known to impact drake reproduction; thus, these factors may help explain the observed variation in fertility. Research on Muscovy ducks identified that drake and hen age could affect reproductive characteristics [50,51]. Furthermore, female duck age is known to influence fertility more than male duck age [50,52]. Egg composition has been correlated with hen age. For example, yolk sac weight increased between Pekin duck hens that were 26 and 31 weeks of age and hens that were 36 weeks of age [50,53]. Since season was confounded with age, we cannot speculate as to whether differences in seasonality affected flock fertility. Fertility in poultry can be affected by a multitude of seasonal factors, including but not limited to variation in humidity, rainfall, temperature, and wind speed [50,54,55].

## 5. Conclusions

Contrary to the predictions of this study, providing male ducklings with physical access to females during rearing did not affect drake mounting behavior or circulating testosterone levels, or flock fertility. Future research should consider other factors experienced during early rearing that may influence reproductive success in commercial ducks, including the influence of auditory and visual cues, possible interactions between duck age, light schedules and/or seasonality, and the impacts of hen choice. Revealing the implications of the observed individual variation in mounting behavior and testosterone levels is another interesting direction for future research. The source of the variation is not clear; neither are the consequences for individual reproductive success of these birds.

## Data Availability

Data is contained within the article or supplementary material.

## Supplementary Materials

The following supporting information can be downloaded at: https://www.mdpi.com/article/10.3390/ani12212979/s1, Table S1: Estimated marginal means and standard error of the mean measurements for correctly oriented mounts/drake/12 h over three days modeled for selected weeks of age for both rearing treatments; Table S2: Estimated marginal means and standard error of the mean measurements for testosterone levels modeled for selected weeks of age for both rearing treatments; Table S3: Estimated marginal means and standard error of the mean measurements for average pen fertility percentages modeled for selected weeks of age for both rearing treatments.
