# Supplementary material for: The Impacts of Female Access during Rearing on the Reproductive Behavior and Physiology of Pekin Drakes, and Flock Fertility
Source: Animals (Basel). 2022 Oct 29;12(21):2979. doi: 10.3390/ani12212979 (PMC9657275; doi:10.3390/ani12212979)
Supplement: Supplementary file 1 [file animals-12-02979-s001.zip › animals-1975641-SI.pdf]

**Supplementary Table S1.** Estimated Marginal Means (EMM) ± SEM for correctly oriented mounts/drake/12 hr modeled for selected weeks of age for both rearing treatments

| Age (weeks) | Same Sex Mounting Events EMM ± SEM | Mixed Sex Mounting Events EMM ± SEM |
|---|---|---|
| 26 | 0.522 ± 0.089 | 0.893 ± 0.134 |
| 32 | 1.61 ± 0.226 | 1.73 ± 0.238 |
| 45 | 1.00 ± 0.153 | 1.10 ± 0.165 |

**Supplementary Table S2.** Estimated Marginal Means (EMM) ± SEM (ng/ml) for testosterone levels modeled for selected weeks of age for both rearing treatments

| Age (weeks) | Same Sex Testosterone Levels EMM ± SEM | Mixed Sex Testosterone Levels EMM ± SEM |
|---|---|---|
| 15 | 0.186 ± 0.014 | 0.166 ± 0.013 |
| 22 | 3.270 ± 0.2557 | 3.277 ± 0.2563 |
| 28 | 7.207 ± 0.564 | 9.615 ± 0.752 |
| 34 | 7.746 ± 0.613 | 8.243 ± 0.661 |
| 45 | 7.267 ± 0.590 | 7.921 ± 0.651 |

**Supplementary Table S3.** Estimated Marginal Means (EMM) ± SEM (%) for average pen fertility modeled for selected weeks of age for both rearing treatments

| Age (weeks) | Same Sex Mounting Events EMM ± SEM | Mixed Sex Mounting Events EMM ± SEM |
|---|---|---|
| 33-34 | 71.6 ± 2.62 | 75.7 ± 2.77 |
| 45-46 | 82.00 ± 3.00 | 86.7 ± 3.17 |
